# The Abnormal Conditions of the Kidneys in Bright’s Disease

**Published:** 1856-10

**Authors:** Austin Flint

**Affiliations:** Professor of Clinical Medicine and Pathology in the University of Buffalo


### THE ABNORMAL CONDITIONS OF THE KIDNEYS IN BRIGHT'S DISEASE.

BY AUSTIN FLINT, M. D.

Professor of Clinical Medicine and Pathology in the University of Buffalo.

In 1827, Dr. Richard Bright, of London, announced the discovery that cases of general dropsy, which the researches of Blackall, Well, Cruikshank, and others had already shown to be frequently associated with the presence of albumen in the urine, are sometimes characterized by a peculiar structural alteration of the kidneys. The validity of this discovery was slowly acknowledged by his professional brethren in the British metropolis. Dr. Christison writing more than ten years after the publication of Dr. Bright's "Reports of Medical Cases," remarks that in a late visit to London, he was surprised to find the doctrines therein contained still questioned by some medical gentlemen. This backwardness, too common in similar instances to occasion surprise, was confined to his own city. Distinguished pathologists in other parts of the British island, and on the continent of Europe, at once recognised a new field in pathological research; and in the absence of definite knowledge of the nature of the disease newly added to the nosological catalogue. it soon became currently known by the title of *morbus Brightii*, or Bright's disease, a term which, if it be not perpetuated, has been already long enough in vogue to preclude the possibility at any period, however distant, of doubt as to the author of the discovery. Among the first to enter upon investigations pertaining to the disease, was Dr. Christison, of Edinburg. He published in 1829, a short paper on the subject, and in 1838, a formal treatise, written with that perspicuity, and pervaded by that candid, philosophical spirit which distinguish all the varied productions of his pen. Dr. Christison designated the disease "Glanular Degeneration of the Kidneys." Papers were also communicated to the Edinburg Medical and Surgical Journal by Dr. James Gregory. In Dublin, the subject was early investigated by Dr. Jonathan Osborne. In Paris, the new disease at once engaged the attention of Rayer, who was at that time, and indeed at the present moment is occupied with researches pertaining to diseases of the kidney; and in the *livraison* of his splendid "*Traite des Maladies des Roins*," issued in 1837, he treats of it at length under the head of albuminous nephritis, (*nephrite albumineuse.*) In the succeeding year, a distinct treatise on the subject appeared in Paris, from the pen of Dr. Martin Solon. During the last twenty years, Bright's disease has held a prominent place in pathological investigations and discussions, wherever pathology is cultivated. Not to attempt an enumeration of the subsequent numerous writers whose contributions have more or less value, Rokitansky of Vienna, Frericks, of Brunswick, Reinhart, of Berlin, and George Johnson, of London, may be mentioned as the most conspicuous among those whose names are identified with the recent and present literature of the subject.

Dr. Bright regarded the affection of the kidneys, which he discovered, as a peculiar species of degeneration, although he describes it as presented under three forms.* In this he is followed by Christison, who recognises the individuality of the disease, and divides it into three stages. Subsequent investigators, however, have ceased to take this simple view of the subject. Dr. Solon divided the affection, after differences in the morbid anatomy, into five varieties. Rayer had already described and figured six. More recently, Rokitansky has extended the number to eight. These diversities are based on the gross appearances of the diseased organs in different cases, and are supposed to represent certain pathological distinctions of greater or less importance. Dr. Johnson, a still more recent writer, taking microscopical appearances as the basis of his division, treats of five different forms of disease, some of which he regards as separated widely by distinctive circumstances pertaining to the seat and nature of the affection. The simplicity of the subject, as presented by Bright and Christison, is in a great measure lost. Late researches, instead of contributing to its elucidation, tend to render it more complicated. The question, "what is Bright's disease?" is at this time not easily answered. The existence of the disease as an unique malady, has been questioned; and by most of the distinguished pathologists last named, the individuality of the affection is virtually denied, the different forms or varieties being, in fact, according to their views, different forms of disease. On the other hand, investigations directed toward the co-existence of a structural affection of the kidneys with the presence of albumen in the urine, and the occurrence of general dropsy, have invalidated the significance of the latter as constant symtomatic phenomena of the disease. Albuminuria and anasarca are found to occur

*It is perhaps proper to state that Dr. Bright's "Report of Medical Cases," published in 1827, is not accessible to me; and any references to his doctrines, as therein presented, will be made on the strength of quotations or statements found in other works.

when an appreciable degeneration of the structure of the kidney has not taken place, and per contra, degeneration of the structure may not be revealed by the excretion of albumen or serious effusion into the areolar structure and cavities. In short, he who undertakes by the study of the literature of the subject, at the present moment, to make himself acquainted with its pathological and practical relations, encounters distinctions, conflicting opinions, and cotroversies, which can hardly fail to occasion perplexity and discouragement. Such I confess to have been my own experience, and it is with a personal sense of the sentiments just mentioned, that I propose, as a topic of inquiry, what are the general views which, with our existing knowledge, the practical physician is to adopt respecting Bright's disease? In the endeavor to this enquiry, the point of view from which I shall survey the subject, is that of a practitioner of medicine who has had some clinical acquaintance with the phenomena embraced under these terms, but who does not profess to have made this branch of pathology the subject of special research. This is the position occupied by the great majority of the medical profession; and none can doubt that the question is an important one. The reality of a grand discovery by Dr. Bright is unquestionable. Numerous affections, it is sufficiently established, are pathologically associated with the disease of the kidney. Now, amidst a diversity of doctrines entertained by those who have given to the matter special attention in the way of original research, what is the physician to recognise as the substratum of his faith and practice? In proceeding to the considerations which are opened up by this question, it is needless to premise that I cannot undertake to enter on a comprehensive analytical review of all the doctrines embraced in the recent literature of the subject. I must confine myself to leading points, and the main object must be to endeavor to determine how far the various topics with which pathologists are occupied, concern the medical practitioner. Surveyed from the general points of view in which the subject is of interest and importance to the medical practitioner, we should be led to consider—1. The abnormal conditions of the kidney in Bright's disease; 2. The significance of the presence of albumen in the urine, or albuminuria; 3. The individuality of the affection discovered by Bright, its symptomatology, associated morbid conditions, sequels, pathology, diagnosis, and treatment. This arrangement covers a broad field of inquiry, and I shall limit myself at present mainly to a circumscribed portion, viz: to the abnormal condition of the kidney.

It would be tedious to reproduce in full the descriptions of the morbid appearances distinguishing the different phases of the disease given by the several pathologists who have been named. A condensed summary of the descriptions by Christison, Rokitansky, Frericks, and Johnson, will embody facts sufficient for our present object.

Dr. Christison, regarding the different appearances as indicating successive periods of the disease, recognises an incipient, middle and advanced stage. In the incipient stage, the kidneys are flabby, friable, more or less enlarged, highly vascular, presenting points and star-like spots of ecchymosis. These are the appearances found in certain cases of acute albuminuria; that is, cases characterised by general dropsy and albumen in the urine, and running rapidly to a fatal issue. The pathological condition in this stage is supposed to be that which precedes the occurrence of a morbid deposit in the kidney. Dr. C., however, admits that the evidence of this is incomplete, and considers it not improbable that in some of these cases the disease is neither more nor less than ordinary nephritis. The middle stage is characterised by a morbid deposit. The kidney, when divided longitudinally, instead of presenting "its usual reddish-brown color and the appearance of coarse striæ in nearly parallel lines, lying in a direction from the centre of the organ towards its surface, is grayish-red, grayish-yellow, or reddish yellow, without any striated arrangement of parallel lines, but of an uniform, sometimes obscurely, sometimes distinctly, granular texture, chequered occasionally with reddish or brownish spots." The deposit, in proportion as it pervades the kidney, apparently supplants the natural structure of the organ. Commencing in the cortical substance, it extends to the portions which dip between the pyramids or tubular cones, and finally may encroach on the latter. The affection thus involves more or less disorganization of the kidneys. The organ, under these circumstances, may be enlarged, preserve its normal size, or undergo some diminution, the breadth of the cortical substance corresponding to these variations. The account given by Dr. C., of the anatomical characters belonging to this stage, depicts appearances which I have repeatedly observed in chronic cases of Bright's disease. In the advanced stage, the disorganization is more complete. The morbid deposit invades the tubular portions, which may be almost entirely abolished. The kidneys now generally suffer reduction in size; in other words, they become atrophied. The surface is rough or lobulated; the texture abnormally firm. Small cavities or cysts are sometimes observed. On longitudinal sections the cortical structure is occupied "by grayish-yellow degeneration, or by a homogeneous substance somewhat like fatty degeneration of the liver." This is an abstract of the more prominent of the gross appearances as described by Christison. His descriptions, by their clearness, simplicity, and obvious fidelity to nature, will repay a careful perusal.

Passing next to Rokitansky, this pathologist, as already stated, describes not less than eight distinct varieties. This redundance of subdivisions, which is a striking feature, if not a serious blemish of his great work on pathological anatomy, renders his account of the morbid appearances rather tedious and complicated. Condensed as much as possible, the distinctive characters of the several varieties are as follows: 1st. The characters which belong to the incipient stage as described by Christison. And Rokitansky states that "we should be obliged to consider the condition as one of very acute simple inflammation of the kidneys, were it not that the characteristic general symptoms and the constitution of the urine established it as a case of Bright's disease." 2. "An infiltration of a grayish or grayish-red, or yellow, viscid and turbid fluid," pervading the organ uniformly or in diffused spots, small punctiform or triated ecchymoses here and there, the tissue presenting a combination of partial anæmia and hyperæmia. The organ increased in size and weight. 3d. Considerable enlargement in size and weight. The cortical substance completely anæmic, tense, friable and infiltrated with a large quantity of opaque, milky white, or yellowish fluid. The superficial layer more particularly, but also the deeper-seated parts, made up of white or yellowish-white granules, (Bright's granulations,) of the size of a poppy seed, or pin's head. The portions of the cortical substance which dip between the pyramids increased and the latter therefore compressed. The granular cortical substance sometimes forcing its way between the tubuli, causing the pyramids to present a frayed or unravelled appearance. The renal fascia easily detached. The mucous membrane of the calyces and pelvis reddened. 4th. Considerable increase in size and weight. The consistency diminished, the tissue very friable and gorged with a large quantity of milky white or yellowish juice. The granulations exceeding the size of millet seeds and equal to that of hemp seeds, and they may project from the surface of the organ. The renal sheath almost unattached. 5th. The kidneys enlarged, or of normal size, or *reduced in bulk.* The surface granular and nodulated, and *in some portions irregularly furrowed and indented, and cicatriform.* The cortical tissue coarsely granulated, very vascular and congested. Cysts containing various substances, and varying in size from that of a poppy seed to that of a pea or nut, scattered here and there. *The attachment of the fascia propria more intimate, and the fascia is thickened.* The pyramids small and atrophied. 6th. The organ but little increased in size and weight. The cortical substance presenting a few undefined patches of a pale color, the prevailing hue pale red, white, yellow, or ashy, and infiltrated with inspissated matter, resembling thick cream or coagulated albumen. *The consistency of texture normal or abnormally increased.* The pyramids, calcyces and pelvis normal. 7th. Trifling increase of size, or partial atrophy. *Density increased.* The cortical substance presents patches of a dull white color without defined borders, arising from a coagulated albuminous lardaceous-looking substance in which no trace of the renal tissue remains. The organ presents the appearance and consistency of fatty cartilaginous tissue. One or more of the pyramids occasionally undergo a similar metamorphosis. *The fascia propria is agglutinated to the diseased portions of the kidney and thickened.* 8th. The kidney of normal size or slightly increased, and considerably indurated. The general hue dirty red or brownish-yellow, and the cortical substance presents a fatty gloss, is unusually hard and brittle, and infiltrated with an albuminous, lardaceous and transparent substance. Occasionally a whitish flocculent deposit is seen in the tissue, of the shape of fine granular dots and lines, giving to the surface and sections a marbled appearance.

It is not easy to obtain a very clear idea of the distinctive characters which distinguish severally these varieties, and the propriety of describing all of them as different forms of the disease, is not apparent, more especially as Rokitansky states that they may be complicated with one another, and since he considers the second, third and fourth forms as representing "progressive stages or degrees of the metamorphosis occurring in Bright's disease." The fifth form he regards as the "last link of the metamorphosis, with it the process becoming retrograde and the disorganized tissue of the viscus, presenting the condition of secondary atrophy." Now on reference to the characters which distinguish the fifth variety from the forms which precede, they are found to be, reduction in the size of the organ, which is not constant but occasional; greater consistency of texture; an irregularly furrowed, indented, cicatriform appearance of the surface, and a more intimate attachment of the fascia propria. These points I have italicised in the abstract of Rokitansky's description. The second, third, fourth, and fifth forms, more particularly represent Bright's disease, and Christison's granular degeneration of the kidney. The sixth and seventh forms represent the less frequent or chronic varieties of the disease, and the latter, (quoting his words,) "must be looked upon as the terminal point of the metamorphosis, as the product of the disease is retained in a state of condensation and organization, and consequently shrivels up." The eighth form, Rokitansky regard as invariably chronic *ab initia*, and springing from inveterate scrofulous or rickety disease, but especially from syphilitic and mercurial taint, and is associated with analogous affections of the spleen and liver, in the shape of lardaceo-albuminous infiltration."

Before proceeding to notice the descriptions by Frericks and Johnson, I shall state more fully the views of the two pathologists just referred to, as regards the morbid process or processes which these appearances involve. The sensible characters constitute the morbid anatomy, but what are the abnormal conditions underlying the visible changes which the organ undergoes? Here will be found differences of doctrine, and these diversities, it is evident, in some instances, have had more or less agency in determining the arrangement of the morbid appearances, if not, indeed, the descriptions of the anatomical characters.

Dr. Christison describes the abnormal changes without any hypothesis as to the process or processes which give rise to them. He says at the outset, that the exact nature of the degeneration is not ascertained. He compares the deposit to that of tubercle. Rokitansky considers Bright's disease to consist in an inflammatory condition. He uses, however, the term inflammation in a wide sense, embracing the process by which "most, and in a certain sense, all general diseases become localized." Tuburculosis, in his view, is equally inflammatory. He also, as already stated, regards the varied anatomical characters in Bright's disease as due to metamorphosis of a morbid exudation in the kidney. The latter idea is more fully developed in the theoretical views of Frericks.

Dr. Frericks published a monograph on Bright's disease in 1851, sustaining a doctrine previously advanced by Dr. Reinhardt, of Berlin, which has the recommendation of reconciling the diversity of anatomical characters with the unity of the disease. The doctrine has met with favor not only in Germany, but, prior to the publication of Dr. Johnson's work, in Great Britain.* He resolves the affection into three stages. The first stage is characterized by congestion and exudation. Under the second stage the exuded matter, which is the plasma of the blood coagulated within the Malpighian tufts and convoluted tubes, undergoes fatty degeneration. The absorption of this fatty matter characterizes the third stage. The fatty matter being absorbed, the kidney is left more or less damaged according to the amount of permanent injury which it has received from the previous changes. The diversity of appearances are explained,

*I am indebted for the account of Frerick's views, mostly to a review by Dr. Johnson in the British and Foreign Med. Chir. Rev., Jan. 1853, and to an article by Prof. J. C. Dalton, Jr., in the Buffalo Med. Journal, March, 1853.

after this theory, by the various circumstances pertaining to the deposit, its transformation and removal by absorption. In the first stage the kidney is enlarged from hyperæmia. The surface of the organ is smooth, and the fibrous capsule more easily stripped off than usual. The deposition is going on in this stage. In the second stage, the organ is still larger than natural, but from the fibrinous exudation, and not from hyperæmia. The appearances due to the latter disappear, and the tissue presents the pale yellow hue distinctive of fatty degeneration. The accumulation of fatty matter in the tubes gives rise to the granulations, and the roughening of the external surface. The tissue in this stage is friable. In consequence of the absorption of the fatty matter in the third stage, atrophy of the organ ensues. And as the process of absorption goes on unequally in different portions, the irregularities on the surface rendering the kidney nodulated, are unaccounted for. More or less wasting of the organ occurs; the texture becomes dense, and the investing capsule adheres closely. To quote from the English reviewer, the following is a summary of the doctrine of Reinhardt and Frericks: "An engorgement of the renal blood vessels, an effusion of inflammatory products, a more or less complete and general metamorphosis of these products into fat, and, finally, atrophy and wasting of the kidney. The small contracted granular kidneys have once been fat; the large, pale kidneys are in continual progress towards atrophy and contraction."

The primary and essential morbid condition, according to Reinhardt and Frericks, is inflammation. The former, indeed, designates the first stage, the *simple inflammatory stage.* Without stopping to comment on the doctrine thus briefly stated, which is certainly attractive from its plausibility and simplicity, we pass to the more recent, and more complicated views of Dr. Johnson.

Dr. Johnson describes several pathological conditions of the kidney giving rise to the symptoms which are supposed to characterize Bright's disease.* He adopts, as the basis of his division, the phenomena furnished by microscopical observations. The reliability of this basis will be the subject of remark presently. The following are the several forms of disease, which he regards as more or less distinct from each other: 1st. Acute desquamative nephritis. 2d. Chronic desquamative nephritis. 3d. Waxy degeneration of the kidney. 4th. Non-desquamative disease of the kidney. 5th. Fatty degeneration of the kidney. The significance of these titles is derived not

*On the Diseases of the Kidney, their pathology, diagnosis, and treatment, with an introductory chapter on the anatomy and physiology of the kidney. By George Johnson, M. D., Lond., etc. London, 1852, 8v., pp. 517.

exclusively from the morbid appearances of the organ, but also from certain abnormal products revealed by the microscope, in the sedimentary deposit in the urine. To render the distinctions intelligible it will be necessary to make some reference to the latter.

1. *Acute desquamative nephritis.*—The size and weight of the kidney are increased. The surface is smooth, and the capsule easily stripped off. Certain portions of the capsular surface and the internal structure are more vascular than natural, and other portions are exsanguine. Ecchymosed spots are observed. The consistence in young and adult subjects is increased, but in aged persons diminished. These appearances accord mainly with those belonging to the incipient stage of Christison, and the stage of engorgement of Frericks, and the first of the eight forms described by Rokitanshy.

Microscopically examined, the convoluted tubes which form a large part of the cortical substance, are more or less filled and distended with epithelial cells, which may sometimes be squeezed out forming "epithelial casts," such as are observed in the sediment of the urine. Some of the tubes are filled with extravasated blood. The vessels constituting the Malpighian tufts are thickened and filled with blood-corpuscles, which are larger and of a lighter color than natural.

The sediment of the urine during life, contains "numerous cylindrical bodies composed of fibrin, which, having exuded from the Malpighian bodies, has coagulated in the tubes, and escaping thence, presents solid cylindrical moulds of the interior of the tubes, in which are entangled blood-corpuscles and epithelial cells which have been shed, by a process of desquamation, from the surface of the tubes." To the casts thus characterized by the presence of recently formed and entire epithelial cells, Dr. Johnson gives the names of *epithelial casts.* Their average diameter is about 1–700 inch.‡

So far as the local affection is concerned, Dr. Johnson regards this form of disease as an acute inflammatory process seated in the lining membrane of the convoluted tubes—a catarrhal affection, as it were, of these tubes.

2. *Chronic desquamative nephritis.*—This succeeds the acute affection, and offers a variety of gross appearances which the author explains by the changes supposed to be discovered by means of the microscope. Diminution in weight and bulk as the disease advances, is a result more constant than any other. The wasting affects first the cortical substance, and a result is

---

‡These casts and the other varieties subsequently mentioned, are figured in Dr. Johnson's work.

the obliteration, more or less complete, of the lobular divisions on the capsular surface. In some instances, the kidney continues to waste and retains its smoothness of surface. In other instances, the decrease in size is less marked, and the cortical substance presents a yellowish-white, firm, and wax-like appearance, attributed to the deposit of the waxy casts, presently to be described, in the convoluted tubes. The vascularity of the organ is least where the deposit is most abundant. The contraction of the deposit gives rise to firm, white granulations varying in size from a pin's head to a pea, by which the capsular surface of the kidney is often roughened.

Cysts are frequently observed of various sizes, but they are comparatively rare in cases of extreme atrophy of the kidney. With reference to these cysts, which have been already embraced in the previous descriptions, Dr. Johnson considers them as formed by dilatation of the convoluted tubes at certain points. On the other hand, Mr. Simon contends that they are "abnormal developments of epithelial germs; his theory being that certain diseases of the kidney tend to produce a blocking up of the tubes; that this obstruction, directly or indirectly, produces rupture of the limitary membrane; and that then, what should have been the intra-mural cell-growth, continues with certain modifications as a parenchytic development." This view is sustained by Rokitansky and Paget. Dr. Johnson devotes considerable space to the discussion of the subject; but it is obviously more interesting as a scientific question, than practically important.

Chronic desquamative nephritis, Dr. Johnson regards as a chronic inflammation of the renal tubes, characterized by a long-continued shedding of epithelium, which appears in the urine in a disintegrated state. "The tubes gradually lose their epithelial lining, and, subsequently, become atrophied, or filled with some new and frequently an unorganized material; or, lastly, they continue to be nourished, secrete serum, into their cavities, and so become dilated into cysts. The kidney in the advanced stages is commonly, but not invariably, much wasted, its substance firm, and its surface irregular."

The sediment in the urine, examined microscopically, is found to contain cylinders, composed of a granular material, which Dr. J. distinguishes by the title of *grnular casts.* The material he regards as disintegraled epithelium. At a later period, other casts are observed mixed with the granular casts. These are of larger size, having a peculiar whitish, waxy appearance, and a well-defined short outline, their diameter being about 1–500 inch. The size of these casts is about the same as that of the renal tubes, and they are considered as having been moulded in tubes which have been deprived of epithelial lining by the desquamative process.

3. *Waxy degeneration of the kidney.*—Certain cases are characterised by the abundance of the waxy casts, just described, in the sedimentary portion of the urine; and they may neither be preceded by, nor associated with, the true desquamation, either in an acute or chronic form. Dr. Johnson considers these cases as constituting a distint form of disease. The cortical substance of the kidney presents an anæmic and pale waxy appearance, forming a strong contrast with the medullary cones. It may be remarked here, that, even assuming the correctness of the basis of Dr. Johnson's divisions, the propriety of making this a distinct form of disease, is certainly questionable. The size of the waxy casts, however, as significant of the absence of epithelium, and denoting an advanced stage in the progress of disease, is both interesting and important.

4. *Non-desquamative disease of the kidney.*—This form is distinguished from the preceding forms, by the absence of the evidence of desquamation of the epithelium of the renal tubes. Cases presenting this distinctive feature, according to Dr. Johnson, may be acute or chronic. In cases of the latter, the kidneys are, usually larger and heavier than natural—the increase in weight exceeding that in bulk. "The most constant and characteristic appearances are gradually increasing wax-like pallor of the cortical substance, and a loss of the color which depends on its vascularity, while the medullary cones retain their vascularity, and have a pinkish color. As the disease progresses, the lobular markings generally become obliterated over a large portion of the capsular surface, until at length there remains only a few small, isolated, vascular patches, as represented in Dr. Bright's fourth plate.*

Hemorrhagic spots are scattered here and there in the cortical substance. The capsule is readily removed. The surface is smooth and ungranulated. The kidney admits of being injected only to a very limited extent.

Microscopically examined, the convoluted tubes generally appear to be simply more opaque than usual. The absence of a morbid deposit within the tubes, is the distinguishing negative feature. Dr. Johnson accounts for the increase of size on the hypothesis of hypertrophy.

5. *Fatty degeneration of the kidney.*—Fatty degeneration of the kidney consists of two varieties, viz: 1. *Granular fat kidney;* and 2. *Mottled fat kidney.* *Granular fat kidney*, Dr. Johnson regards as in some cases eventuating from the form last noticed, viz: non-desquamative disease, and in other cases

*The lobular divisions of the kidney in health, are pentagonal or hexagonal in form, and average about one-eighth of an inch in diameter. They bear an analogy to the lobules of the liver.

succeeding acute desquamative inflammation. The kidneys present the general characters just described as belonging to non-desquamative disease. Their size and weight are increased, the surface is smooth, and the cortical portion is pale and non-vascular. They are characterised, in addition, by yellowish-white granulations, which resemble the so-called atheromatons patches in arteries, are found, on a microscopic and chemical examination, to be composed almost entirely of fatty matter. A large proportion of the convoluted tubes, under the microscope, present the same appearance as the non-desquamative form last described; but some contain the white wax-like material, and occasionally some are filled with blood, giving rise to hæmorrhagic spots which are visible by the naked eye. The characteristic appearance just mentioned of this form of disease is due to the presence of oil-globules in a greater or less proportion of the tubes. The oil is formed in the epithelial cells, and it exists in abundance in proportion to the progress made in the process of fatty degeneration. As this process advancess, the cells assume a globular or oval form, and when completely filled with oil-globules they appear opaque and dark. Rupture of the tubes frequently takes place from over-distension by the enlarged cells containing oil. The oily or fatty nature of material forming the yellow granulations visible with the naked eye is shown, first, by the peculiar and characteristic appearance of the globules when examined by the microscope; and secondly, by the fact that both the granulations seen by the naked eye, and the globules visible by the microscope are entirely removed by digestion in ether. The fatty granulations may be few in number, or thickly disseminated through the cortical substance. Dr. Johnson admits that some of the oil is a product of a metamorphosis of a fibrinous or albuminous effusion into the tubes, but he thinks a small portion only has this origin, the greater part being contained in distinct cells.

The sediment of the urine in this form of disease is found to contain small waxy casts, in which are entangled a few globular or oval cells enclosing a variable number of oil-globules. This constitutes a distinctive trait of the present form of disease, contrasted with that last noticed, which is ascertained during life.

In *mottled fat kidneys*, the organ is usually increased in size and weight; the color of the cortical substance is either uniformly pale, or more frequently pale, or more frequently mottled by red vascular patches, and occasionally hæmorrhagic spots are observed; the medullary cones retain their natural color and vascularity; the kidney is usually softer than natural. Microscopically examined, everywhere the convoluted tubes are found to be greatly distended with oil, which has accumulated in their epithelial cells. The oil-globules are of a larger size than in the granular fat variety. The yellow granulations visible by the naked eye in the former variety, are not apparent in this, these granulations being the result of certain tubes distended with oil, while the surrounding tubes are nearly or quite free from this material. In the mottled variety, the peculiar appearance arises from the convoluted tubes being almost invariably and equally filled with oil. In this variety, a large quantity of oil is obtained from the kidney by chemical analysis; even so large a proportion as $\frac{1}{4}$ of the solid substance has been found to consist of this matter.

Advanced fatty degeneration, in the opinion of Dr. Johnson, is the most hopeless form of renal disease. And when this condition is indicated by urine highly albuminous, and by the presence of numerous oily casts and cells in the sedimentary deposit, he regards the malady as not less serious and intractable than tubercular disease of the lung. In waxy degeneration and non-desquamative disease, the prognosis is less favorable than in the acute or chronic variety of the desquamative form.

Assuming the correctness of the observations of Dr. Johnson, (and certainly I am not prepared to question their accuracy,) it is obvious that the foregoing classification rests measurably on speculative ground. Without specifying points purely hypothecal, which will suggest themselves to the reader, the adequateness of microscopical appearances to serve as a basis of the pathological distinctions involved in his division and descriptions, is inadmissible. By this remark, I am far from intending to disparage the microscope in researches pertaining to morbid anatomy. Its great value in this relation is unquestionable. And with respect to the abnormal conditions of the kidney in Bright's disease, its utility is not to be denied. But to predicating the anatomical characters of disease solely, or even mainly on the revelations of the microscope, there is this serious if not insuperable objection, viz: the examination cannot, without an immensity of labor, embrace the totality of the organ or part diseased, but must be limited to extremely minute portions. This difficulty is well expressed in the following quotation from a paper by Dr. T. K. Chambers, on the subject of the present article. "In microscopic researches, no count can be kept of what is, practically speaking, one of the most important elements of pathological classification —namely, the extent of distribution of morbid processes. The existence of a particular diseased state in the small peice or pieces which are submitted to the lens may be easily made evident; but whether this diseased state is an accidental local circumstance, or is spread through the organ in such amount as probably to interfere with its functions can only be judged by the naked eye. To set down microscopic deviations from health as *the* disease would lead to mistaken views. For example, the hearts of few chronic invalids can be examined without some part of the muscle exhibiting that granular condition which is found when the fibre is about to become oily, but to designate all these hearts as instances of fatty degeneration, and to suppose that they had any calculable influence on the duration of life, as distint from the general diathesis, would surely be a serious error. Yet such, I think, would be the result, risked by adopting microscopic appearances as a principle of pathological division."*

We come now to the question, What are the ascertained facts respecting the abnormal conditions of the kidney in Bright's disease, which it behooves the physician to know and recollect? In other words, what are the points pertaining to the descriptions and distinctions just passed in review, which are satisfactorily established, and which possess practical importance? Eliminating all that is doubtful or superfluous, and it will be found that observations since the publications of Bright and Christison have not added much that is positive and practically useful to our knowledge of the morbid changes incident to the disease. I shall attempt to embody in a few words, the sum of what is actually known of these changes, in so far as concerns their nature, seat and pathological relations.

1. It is abundantly established that albuminuria may exist together with anasarca and other symtomatic phenomena, and death take place without a morbid deposit in the kidney sufficient to give rise to an appreciable alteration in its gross appearances. In certain cases it is to be found to be simply more or less engorged, enlarged, and friable, the capsule easily detached owing to serous effusion into the subjacent areolar tissue; and in some instances, even these morbid characters are wanting, the organ appearing to be healthy. Dr. Johnson gives a case exemplifying the fact last stated, under the head of "non-desquamative disease of the kidney." In most of such instances, according to Dr. Johnson, microscopical examinations would be expected to reveal morbid conditions of the renal epithelium. But his own observations show that if this be the rule, it is not without exceptions. And these exceptions render it extremely doubtful whether Dr. Johnson be correct in regarding the epithelium of the convoluted tubes as the point of departure of the local affection in the cases included under the head of "desquamative nephritis." To deny that the evidence of desquamation

*Decennium Pathologicum. Brit. and For. Med. Chir. Rev, April, 1853.

of the renal epithelium is afforded during life and after death in some cases and not in others, would be to question the accuracy of Dr. Johnson's observations. But it may fairly be doubted whether this constitutes a radical pathological distinction, and the propriety of the nosological division into desquamative disease is therefore questionable. The simple fact is, that in certain of the cases characterized by the phenomena of Bright's disease, the sediment of the urine abounds in casts, and they are found in the convoluted tubes after death, while in other cases the urine is free from casts and none are discovered in the tubes.

It may be remarked here, that the existence of Bright's disease as determined by the symptoms during life, without any appreciable lesion of the kidney being apparent after death, is a strong argument in faver of the doctrine that the essential pathology of the affection involves blood-changes anterior to those occurring in the kidney. In these cases the disease runs a rapid course. They are cases of *acute* albuminuria; and it may be presumed that the fatal termination occurs too quickly for those morbid alterations in the structure of the kidneys to take place which are generally found in chronic cases. The opinion held by most pathologists is, that prior to the lesions due to a morbid deposit, for a greater or less period the only appreciable morbid condition is a state of congestion or hyperæmia; in other words, that this condition is the first link in a chain of sequences. However probable this may be, it is only an inferential conclusion, not an ascertained fact.

2. In most chronic cases presenting the symptomatic phenomena charcteristic of Bright's disease, structural lesion of the kidneys is manifest. The appearances are by no means uniform, as has been seen in the numerous varieties described by distinguished pathological observers. In accounting for the diversity of morbid changes, there is scope for speculation, and it is important to discriminate between that which is ascertained and that which is hypothetical. A just ground for a division is the existence, or not, of atrophy of the kidney. The organ may be increased in bulk, or retain its normal size on the one hand, and on the other hand, its volume may be more or less diminished. What are the facts pertaining to the non-atrophous condition? The appearances are due, directly or indirectly, to the presence of a morbid deposit, doubtless primarily taking place within the convoluted tubes and capsules of the Malphighian bodies which form the cortical substance of the organ. The deposit occurs first in the peripheral portion of the cortex, and progressively advances from the surface to the centre, extending at length between the tubular cones, and even between the individual tubuli, and finally encroaching more or less on the space which belongs to the medullary portion of the organ. The character of this deposit, as studied in different cases, differs considerably. It may consist of coagulated fibrin, with or without blood corpuscles; accumulated epithelium, oil globules and granular fat—these varieties being either separate or more or less combined. The gross appearances will differ according to the character of the deposit, the extent of its diffusion, and its abundance in the portions affected. The size of the organ, its color, its consistence, the irregularity of its surface or its smoothness, the absence or presence of the lobular markings, etc., will depend on the variations just referred to in connection with local hyperæmia or anæmia, the latter resulting in a great measure from the presence of the deposit. These are facts pertaining to the morbid anatomy of the disease. Now as to the relations of the different forms of deposit to each other, and still more their relations severally to ulterior pathological conditions, opinions, with our present knowledge of the subject, must be speculative. It may fairly be doubted whether we are authorized to base on these diversities of appearance important pathological distinctions. Whether the exudation of fibrin be the primary step in the processes of structural alteration, and the detachment of epithelium be simply a mechanical result, according to Frericks; or whether the epithelial cells first become affected, and are shed in more or less abundance as a consequence of an effort to eliminate a morbid material from the blood, as contended by Johnson, must be considered a question open for discussion and farther investigation. So with respect to the different kinds of deposit; do they severally preserve their peculiar character, or do they succeed each other? This question has an important bearing on another, viz: whether the various morbid conditions grouped under the title of Bright's disease, are different stages of a single affection, or, in fact, constitute different affections? Assuming the latter to be true according to the theory of Reinhardt and Frericks, does fatty degeneration occur by *conversion* as contended for by the authors just named, and also admitted in part by Johnson, or must this change always of necessity take place by *substitution* in this as well as other situations, on the principle adopted by Robin and others, that protein substances never undergo metamorphosis into fat? These are questions of scientific interest, which we must at present consider as unsettled. So, also, with respect to the nature of the morbid action which gives rise to the deposit, is it, or is it not inflammatory? In other words, does Bright's disease involve nephritis? This question belongs in the same category as the foregoing. If the fact of fibrinous exudation be sufficient evidence of inflammation, then, unquestionably, this process is often involved; but the inquiry arises whether, in view of the capillary vessels which form the Malphighian tufts, lying loose and uncovered within the dilated extremities of the convoluted tubes, it is not quite probable that mere congestion may lead to the effusion of the liquor sanguinis in this situation.

There are various morbid appearances which are evidently due to merely accidental circumstances. Such are the stellated spots of ecchymosis, the cysts ascertained by the microscope, and also by the naked eye, attaining in some instances to a considerable size, and inflammation of the mucous membrane lining the calyces and pelvis. I mean by the term accidental that they have no necessary connection with the local morbid conditions belonging to the disease. In this respect they differ from changes incidental to a morbid deposit, such as increased breadth of the cortical substance, friability, anæmia, etc.

3d. The foregoing remarks have had reference to non-atrophous kidney. A marked reduction in size constitutes in itself a highly distinctive feature of a class of cases of Bright's disease; but it is associated with peculiar morbid appearances pertaining to the form and the internal structure of the organ. The atrophy not going on equally in every part of the kidney, gives rise to an uneven lobulated surface; to the granulated aspect, the cicatriform furrows, and the puckerings which are observed under these circumstances. The texture becomes hard and resisting. The capsule adheres with an unnatural firmness, so that in peeling it off small portions of the substance of the kidney are torn way with it. The wasting affects primarily and chiefly the cortical portion which is sometimes reduced to an extremely thin layer, the normal structure, in fact, entirely disappearing.

Pathologists generally are agreed in regarding these changes as consecutive to those which belong to the non-atrophous condition. The morbid deposit first supplants the normal structure; and, in fact, it induces atrophy of this structure, while the bulk of the organ (owing to the presence of the deposit) is not diminished. The reduction in size takes place as the result of the elimination of the morbid deposit within the tubes, possibly its removal in a measure by absorption, together with the contraction or carnification of portions which remain. Many interesting questions are involved in the mechanism of these alterations; but, with our present knowledge, they would lead only to opinions and hypotheses, not to fixed facts of science.

In attempting to determine by means of an anlysis, and a comparison of the observations of different pathologists, the sum of our actual knowledge of the normal conditions of the kidney in Bright's disease, we are led to the conclusion that the subject is still shrouded in confusion and difference in doctrine. But this is not surprising; nor does the fact militate against the individuality of the disease. To cite an illustration which has been used for this purpose by more than one writer, until quite lately the morbid anatomy of tuburculosis of the lungs offered not less scope for doubts, discussions and diversity of opinion. Even more important questions remain to be settled. The nature of tuburcle, its primary seat, the local process upon which the exudation depends, its dependence on a peculiar diathesis, the changes which the deposit undergoes, the mode in which they are effected, the relations of the different forms of deposit to each other, etc., etc., are points of scientific interest and practical importance, which are becoming understood in consequence of continued investigation. But, notwithstanding, they have not been heretofore, and are not yet, in all respects, explained, the existence and the unity of the disease called pulmonary tuburculosis, has not been questioned. So with regard to the morbid appearances of the kidney in Bright's disease; when the legions on which they depend are more fully known, their mutual relations will be apparent, and it will probably be more evident than at present, that their pathological origin is not in the kidney, but that the changes in this organ constitute the local expression of a peculiar dyscrasia.

Passing from the consideration of the morbid anatomy of the disease to its pathological consequences and clinical history, we enter on a field more satisfactory and attractive. The obscurity which hangs over the anatomical characters does not pervade, to the same extent, the immediate effects of the structural lesions. These effects consist, 1st, of the transudation into the uriniferous tubes of certain of the elements of the blood; and, 2d, of a deficiency or absence in the urine of certain important constituents of that excretion. Taking these immediate effects as a point of departure, and determining remote consequences by means of clinical observations, the study of the disease becomes at once invested with great interest as well as importance to the physician. We may resume the subject at some future time, with hope of making some amends to the reader, for the tedious and dry details which necessarily belong to the consideration of the branch to which our attention has been restricted in this article.—*Louisville Review.*

